# Validation of the short version of Cognitive Emotion Regulation Questionnaire for adolescents in Norway

**DOI:** 10.1177/14034948231225616

**Published:** 2024-02-01

**Authors:** Sjur S. Sætren, Wenche ten Velden Hegelstad, Tore Tjora, Gertrud S. Hafstad, Else-Marie Augusti

**Affiliations:** 1 Norwegian Centre for Violence and Traumatic Stress Studies, Department for Child and Adolescent Research, Norway; 2 TIPS Centre for Clinical Research in Psychosis, Stavanger University Hospital, Norway; 3 Institute of Social Studies, Faculty of Social Sciences, University of Stavanger, Norway

**Keywords:** Emotion regulation, adolescents, CERQ, validation, population-based study

## Abstract

**Background/Aim:**

The Cognitive Emotion Regulation Questionnaire (CERQ) is among the most popular and widely used measures of emotion regulation across age groups. This study aimed to validate the CERQ short version (CERQ-short) for use on adolescents in the Norwegian population.

**Method:**

A sample of 3461 adolescents (47.3% girls) aged 12–16 years was recruited through the UEVO population-based study of child maltreatment in Norway. Factor structure, reliability, measurement invariance and criterion validity were investigated.

**Results:**

Confirmatory factor analysis supported the original nine-factor model including 18 items; however, not a two-factor structure nor a higher order two-factor solution. Internal consistency was adequate for all subscales, with alpha levels ranging from .73 to .84 between subscales across genders. Relationships with internalizing problems measured with the 10-item Hopkins Symptom Checklist and health-related quality of life according to KIDSCREEN-10 supported the criterion-related validity of the Norwegian CERQ-short.

**Conclusions:**

**Results suggest that the CERQ-short can be used to measure cognitive emotion regulation strategies in the Norwegian adolescent population. The validation of the CERQ-short in Norway could significantly improve mental health care by facilitating better diagnosis, treatment planning, and evaluation, as well as informing public health policy and cross-cultural research**.

## Introduction

Emotion regulation can be defined as the conscious (explicit) or unconscious (automatic) process of goal-directed monitoring, evaluation and modulation of emotional responses [1]. It influences when and how emotions are experienced, their intensity and, in turn, their expression to others. A particularly important component in emotion regulation concerns cognition. Emotion regulation is contingent upon the cognitive appraisal of emotionally salient stimuli, followed by the implementation of strategies that facilitate the modulation and utilization of emotional responses in complex environmental contexts [2,3]. To regulate emotions efficiently, an individual may apply a broad set of cognitive emotion regulation strategies responding to negative or stressful events. Some of these strategies, such as positive reappraisal or refocusing on planning, are considered adaptive in modulating succeeding emotional responses, and help to adapt to difficult situations efficiently [4]. Habitual use of strategies such as self-blame, rumination or catastrophizing are maladaptive and are associated with negative and overwhelming emotional responses. They can confer significant disturbances in daily functioning and have been linked to internalizing psychopathology in terms of anxiety and depression [2,4–6]. Emotion regulation is regarded as an important and trans-diagnostic process in the development and treatment of psychopathology [7].

The Cognitive Emotion Regulation Questionnaire (CERQ) is among the most widely used measurements of emotion regulation [8]. CERQ was developed in 1999 to capture the cognitive strategies of emotion regulation [9]. The original 36-item version captures nine cognitive emotion regulation strategies: *Self-blame*, *other-blame*, *rumination*, *catastrophizing*, *acceptance*, *positive refocusing*, *refocus on planning*, *positive reappraisal*, and *putting into perspective* [9,10]. A shorter version (CERQ-short), comprising 18 items, was later developed in order to increase usefulness for clinical work and for research on emotion regulation in larger population-based studies [10]. The CERQ-short saves time, which is more convenient for respondents, and increases data gathering efficiency. Despite being shorter, it maintains the psychometric quality of the full CERQ [10]. It makes a versatile tool for diverse applications, including clinical trials and population surveys. A nine-factor structure has been consistently supported for the original CERQ [11] as well as the CERQ-short [10]. However, an alternative two-factor structure has also shown acceptable fit indices [12]. This structure organizes the different cognitive emotion regulation strategies into adaptive and maladaptive strategies.

Due to its high psychometric quality, the CERQ-short has been translated and validated for use in different countries and populations [13,14]. Further, it has been found to be consistent across age and gender in adolescents [15,16]. Gender differences in the use of specific strategies have been identified in several studies [17,18], underscoring the importance of evaluating gender invariance in psychometric studies.

Previous psychometric studies have produced consistent evidence for clinical accuracy in terms of criterion validity for both the CERQ full and short versions. Higher scores on the subscales indicating maladaptive cognitive emotion regulation (i.e. self-blame, other-blame, rumination, and catastrophizing) are associated with internalizing problems in terms of depressive symptoms and anxiety [5,17,19]. Clinical samples score higher on items of self-blame, rumination and catastrophizing, while non-clinical samples report greater use of positive reappraisal and putting into perspectives [5,20]. Furthermore, associations between self-blame, rumination and catastrophizing and symptoms of anxiety and depression have been supported across countries, suggesting maladaptive cognitive emotion regulation as a trans-cultural factor related to psychopathology [6]. Inversely, adaptive emotion regulation strategies such as positive reappraisal, refocusing, planning and acceptance appear to be negatively associated with psychopathology [6,21] and positively with quality of life [22,23]. In sum, studies support the construct validity and reliability for CERQ across countries, while it still needs an evaluation for use in the Norwegian adolescent population.

The current study aimed to investigate the psychometric properties of the CERQ-short [10] within a nationally representative sample of adolescents in Norway. The primary objectives were to evaluate the applicability of the nine-factor model and an alternative two-factor model of the CERQ-short among adolescents in Norway, assess its consistency across age and genders by exploring potential age and gender invariance. Furthermore, we aimed to examine internal consistency. Additionally, we tested the criterion-related validity of the CERQ-short subscales by examining their associations with quality of life and internalizing problems, specifically anxiety and depression.

## Method

### Participants

Data for the present study were derived from the Norwegian Youth Study on Child Maltreatment (The UEVO study [24]). The UEVO study is a population-based study of adolescents’ exposure to child maltreatment in a Norwegian population representative sample based on stratified sampling. Data collection took place in two follow-up waves during 2020 and 2021. The current data were drawn from the third and last wave. The initial sample comprised 3540 (47.3% girls; *n* = 1675) adolescents aged 12–16 years recruited through 35 schools in Norway. The response rate was 71.9%. As the survey was in Norwegian, adolescents with insufficient mastery of the written language were excluded from participation.

### Measures

#### CERQ-short

CERQ-short consists of 18 items measuring habitual use of specific cognitive emotion regulation strategies in response to difficult or threatening life events [10]. It includes the same nine subscales as the original 36-item version: *self-blame*, *other-blame*, *rumination*, *catastrophizing*, *acceptance*, *positive refocusing*, *refocus on planning*, *positive reappraisal*, and *putting into perspective*. Five subscales represent adaptive, and four represent maladaptive, regulatory responses. Each subscale consists of two items, each presenting a statement to which participants rate applicability from 1 ‘Almost never’ to 5 ‘Almost always’.

The Norwegian CERQ-short was developed based on the original English version and was translated into Norwegian following the back-translation method [25] by a professional translator. To ensure preservation of content, two independent native speakers of both Norwegian and English evaluated the translation during both phases of the translation process.

#### Internalizing problems

Anxiety and depression were measured using the Norwegian version of the Hopkins Symptom Checklist (HSCL-10 [26]). It assesses symptoms of depression and anxiety on 10 items presented on a four-point scale from ‘Not at all’ to ‘Extremely’. A total mean score is calculated ranging from 0 to 3, where higher scores indicate worse symptoms. The HSCL-10 has high internal consistency [26], with a reported Cronbach’s alpha of .91 in the present sample. The scale has previously been validated for the screening of internalizing problems in adolescents between the ages of 14 and 16 years in Norwegian primary care institutions [26].

#### Quality of life

A Norwegian version of the KIDSCREEN-10 was used to assess adolescents’ self-reported health-related quality of life [27]. The KIDSCREEN-10 includes 10 items reflecting quality of life (e.g. item 1 ‘Have you felt fit and well?’). All items are presented on a five-point response scale, where responses on items 1 to 9 are presented from (1) ‘Not at all’ to (5) ‘Extremely’, and item 10 is presented from (1) ‘Never’ to (5) ‘Always’. In line with the test manual, responses are scored and interpreted as standardized *t*-values, with a mean value of 50 and a standard deviation of 10. Higher scores indicated better quality of life. Previous psychometric evaluation has supported adequate reliability and validity the use in population-based research [27], with Cronbach’s alpha of .84 in the present sample.

### Statistical analysis

Descriptive statistics, reliability- and criterion-related validity analyses were performed using SPSS version 27 [28]. Internal consistency of the CERQ-short, as an index of reliability, was estimated by calculating Cronbach’s alpha coefficients for the total sample and for each gender (male/female) separately. We evaluated gender differences by performing a series of independent sample *t*-tests on the CERQ-total score, HSCL-10 and KIDSCREEN, and reported the effect size using Cohen’s *d*. The same process was also applied to each subscale for CERQ-short.

Confirmatory factor analysis (CFA) and multi-group CFA (across age and gender) were estimated using Mplus [29]. We conducted CFA to test the latent structure of the CERQ-short, including a nine-factor model, a two-factor model and an alternative higher order two-factor model [15,30]. Weighted least square mean and variance adjusted were used as method of estimation, which have some robustness for not-normally distributed variables. All variables except for items 14, 17 and 18 were normally distributed (skewness <±1; kurtosis <±3). In line with recommended fit indices [31], the chi-square index (χ^2^) is reported, and the goodness of fit of the CFA was tested using root mean square error of approximation (RMSEA), comparative fit index (CFI) and the Tucker–Lewis index (TLI) [31]. Acceptable fit was determined by an RMSEA <.06, CFI score >.95 and TLI score >.90 [31]. Threshold values for interpretation of factor loadings were as follows: .32 (poor), .45 (fair), .55 (good), .63 (very good) or .71 (excellent) [32,33].

We conducted a series of multi-group CFAs to investigate measurement invariance across gender and age, separately. For gender, we treated boys and girls as two groups. Regarding age, we considered three age groups: 12 and 13 years, 14 years, and 15 and 16 years. Three different aspects of invariance were considered, including configural invariance (Model 1), metric invariance (Model 2) and scalar invariance (Model 3). Configural invariance examines whether the factor structure is equivalent across groups, where intercepts and residuals of observed variables together with all estimates are freely estimated. Metric invariance examines the equivalence of the magnitude of factor loading to ensure similarity of the loadings across groups. Scalar invariance tests the invariance of item thresholds and indicates the degree to which mean levels of the latent construct are similar across groups. Each procedure relies on the preceding condition being met. Changes in CFI and TLI were used to evaluate invariance; ΔCFI ⩽.010 [34,35]. Significance test of chi-square comparisons between models were also conducted [34].

Criterion validity, meaning how well the scale score correlates with an established outcome it was designed to measure, was evaluated estimating Pearson correlations between the CERQ-short subscales (i.e. self-blame, acceptance, rumination, positive refocusing, refocus on planning, positive reappraisal, putting into perspective, catastrophizing and other-blame) and the HSCL-10, the latter providing an indication of internalizing problems (i.e. depressive and anxiety symptoms). This was also conducted in relationship with health-related quality of life as indicated by KIDSCREEN-10. Correlation analyses were controlled for age and gender. This is in line with previous validation studies (e.g. Garnefski and Kraaij [10], Orgilés et al. [36]).

### Procedure

Data were collected through a secure web-based survey, conducted within a specified time frame at school and during school hours. For a full description of the survey and procedure see our Cohort Profile for the original study [24]. We presented all participants with a 5-min information video to ensure a correct understanding of the assessment procedure, and to increase participants’ motivation. The video also explained the ethical principles of voluntary participation, confidentiality and the right to withdraw from the study at any time without reason. Privacy was ensured by strict guidelines regarding the physical space with 1 m between participants during data collection. All participants were recommended to turn down the light of their computer screen. The survey was constructed so that all answers disappeared from the screen after their response. All teachers were instructed to not go around the classroom and stand behind their students during the survey. The survey took approximately 30 min to complete.

### Ethics

The Regional committee for ethics in medical and health research in the Southeastern region of Norway (Case #2018/522) approved of the study protocol. All participants gave written informed consent. In accordance with a regulation added to the Norwegian Health Research Act in 2018, adolescents did not need parental permission to participate.

## Results

### Descriptive statistics

Table I details descriptive statistics for sample characteristics. The final sample consisted of 3461 out of the original 3540 adolescents (47% girls; *n* = 1677; missing = 24). Exclusion was due to incomplete data comparison on the CERQ-short, HSCL-10 and KIDSCREEN-10 for 79 participants. Mean age was 14 years of age with a range from 12 to 16 years, both in the total sample and across both genders. For further analyses we separated age into three groups: 12 and 13 years (*n* = 564), 14 years (*n* = 1026) and 15 and 16 years (*n* = 1608). Twenty-six per cent reported having a parent from outside the Nordic (Norway, Sweden and Denmark, Finland and Greenland) region. Visual inspection and test statistics indicated normally distributed mean scores for CERQ-short and KIDSCREEN (within the range of ±1.0). HSCL-10 mean scores were positively skewed (1.1).

**Table I.** Descriptive statistics.

Variables	Total (*N* = 3425)		Female (*n* = 1784)		Male (*n* = 1677)		Cohen’s *d*
	*M*	SD	*M*	SD	*M*	SD	
Age (12–16)	14.48	0.96	14.47	0.97	14.49	0.95	–.01
HSCL-10 (0–3)	0.69	0.73	0.94	0.77	0.42	0.55	.77**
KIDSCREEN (*t*)	46.26	10.36	43.59	9.12	49.13	10.69	–.55**

KIDSCREEN scores = standardized *t*-values.

** *p* <.01.

HSCL-10: 10-item Hopkins Symptom Checklist

Significant gender differences were observed for internalizing problems (HSCL-10; *t* (3313) = 22.374, *p* < .001) and quality of life (KIDSCREEN; *t* (3210) = −15.769, *p* < .001). For CERQ subscales, analysis of gender differences revealed some variations. Females significantly reported greater levels of self-blame (*t* (3079) = 4901, *p* < .001), rumination (*t* (3095) = 4717, *p* < .001), positive refocusing (*t* (3041) = 2232, *p* < .001), putting into perspective (*t* (2952) = 5380, *p* < .001) and catastrophizing (*t* (3026) = 7330, *p* < .001), as delineated in Table II. On the other hand, males exhibited significantly higher reports of acceptance (*t* (2958) = −6757, *p* < .001) and positive reappraisal (*t* (3086) = −4889, *p* < .001). All effect size can be interpreted as small (*d* = .2 or smaller) and the subscales refocus on planning (*t* (2958) = −1684, *p* = .093) and other-blame (*t* (2980) = −1227, *p* = .220) yielded no significant differences between genders.

**Table II.** Internal consistency, mean and SD for subscales of the CERQ short version.

CERQ subscales	Total		Female		Male		Cohen’s *d*
	α	Mean/SD	α	Mean/SD	α	Mean/SD	
Self–blame	.77	1.96/0.99	.78	2.11/1.04	.74	1.93/0.97	.17**
Acceptance	.85	2.71/0.88	.83	2.53/1.11	.86	2.82/1.29	–.24**
Rumination	.78	2.41/1.13	.77	2.34/1.06	.78	2.16/1.11	.16**
Positive refocusing	.83	2.33/1.13	.85	2.34/1.17	.81	2.25/1.13	.08*
Refocus on planning	.77	2.38/1.08	.76	2.27/1.02	.77	2.34/1.10	–.06
Positive reappraisal	.76	2.56/1.16	.74	2.40/1.10	.77	2.60/1.21	–.17**
Putting into perspective	.79	2.41/1.12	.81	2.50/1.16	.78	2.28/1.13	.19**
Catastrophizing	.78	1.79/0.99	.79	1.85/0.98	.76	1.60/0.86	.26**
Other-blame	.74	1.88/0.88	.74	1.78/0.86	.73	1.82/0.85	–.04

α = alpha level.

* *p* < .05

** *p* < .01.

CERQ: Cognitive Emotion Regulation Questionnaire

### Reliability, mean and standard deviation

Table II outlines means and standard deviations for CERQ-short subscales for the total sample and for each gender separately. Reliability analyses indicate adequate internal consistency for all nine subscales in both the total sample and across genders (α > .70), with alpha values ranging from .73 (other-blame for boys) to .84 (acceptance for boys).

### CFA

Data supported the nine-factor model (χ^2^ = 1349.237, *p*<.001, degrees of freedom = 99). The model fit was excellent, as indicated by all fit indices: CFI = .974, TLI = .959, RMSEA = .063 (90% confidence interval (CI) = .060–.066). As shown in Table III, item loadings for the nine-factor model were all interpreted as excellent [32,33], ranging between .78 and .95. Results did not support a two-factor model as indicated by all fit indices: CFI = .763, TLI = .729, RMSEA = .162 (90% CI = .160–.165). Additionally, we did not find support for the alternative higher order two-factor structure (CFI = .939, TLI = .925, RMSEA = .085 (90% CI = .083–.088).

**Table III.** Standardized factor loadings for the confirmatory factor analysis of CERQ-short.

Factor	Item	Estimate
Self-blame	I feel that I am the one who is responsible for what has happened	.81
Self-blame	I think that basically the cause must lie within myself	.86
Acceptance	I think that I have to accept that this has happened	.84
Acceptance	I think that I have to accept the situation	.95
Rumination	I often think about how I feel about what I have experienced	.83
Rumination	I am preoccupied with what I think and feel about what I have experienced	.87
Positive refocusing	I think of pleasant things that have nothing to do with it	.89
Positive refocusing	I think of something nice instead of what has happened	.87
Refocus on planning	I think about how to change the situation	.80
Refocus on planning	I think about a plan of what I can do best	.86
Positive reappraisal	I think I can learn something from the situation	.87
Positive reappraisal	I think that I can become a stronger person as a result of what has happened	.78
Putting into perspective	I think that it hasn’t been too bad compared to other things	.85
Putting into perspective	I tell myself that there are worse things in life	.85
Catastrophizing	I keep thinking about how terrible it is what I have experienced	.83
Catastrophizing	I continually think how horrible the situation has been	.89
Other-blame	I feel that others are responsible for what has happened	.80
Other-blame	I feel that basically the cause lies with others	.85

All factor loadings were statistically significant with *p* < .001.

CERQ-short: Cognitive Emotion Regulation Questionnaire short version

### Measurement invariance across genders

We tested the factorial invariance for only the nine-factor model as the two-factor solution was not supported. As shown in Table IV, results suggest that CERQ-short has an excellent fit for both males and females. Multi-group CFA revealed that measurement invariance across males and females was supported. All fit indices for the configural, metric and scalar equivalence testing were satisfactory (see Table IV). The chi-square comparison of configural, metric and scalar invariance suggested significant differences between the configural and the metric model, and between the metric and the scalar model (*p* < .05). CFI difference for metric and scalar equivalence were acceptable (smaller than ⩽.010 [34,35]). Taken together, this suggests that the Norwegian version of the CERQ-short shows measurement invariance across genders, meaning that the factor structure is stable across genders.

**Table IV.** Goodness-of-fit indices and model comparisons for measurement invariance across gender and age.

Model	χ^2^	df	TLI	CFI	RMSEA	(90% CI)	Model comparison χ^2^		ΔCFI
Females	610.013*	99	.964	.977	.058	(.053–.062)	–	–	–
Males	838.557*	99	.955	.971	.068	(.064–.073)	–	–	–
1	1450.500*	198	.959	.974	.063	(.060–.066)	–	–	–
2	1467.871*	207	.961	.974	.062	(.059–.065)	2 *vs*. 1	24.605*	.000
3	1459.501*	252	.969	.975	.055	(.052–.058)	3 *vs*. 2	114.990*	.001
12 and 13 years	298.029*	99	.963	.976	.060	(.052–.068)	–	–	–
14 years	541.470*	99	.952	.969	.066	(.061–.071)	–	–	–
15 and 16 years					.063				
1	1531.037*	297	.960	.974	.062	(.059–.066)	–	–	–
2	1537.377*	315	.963	.974	.060	(.057–.063)	2 *vs*. 1	17.719	.000
3	1469.621*	405	.975	.978	.050	(.047–.052)	3 *vs*. 2	102.814	.004

Model 1 = configural invariance; Model 2 = metric invariance; Model 3 = scalar invariance.

* *p* < .05

χ^2^: chi-square; df: degrees of freedom; TLI: Tucker–Lewis Index; CFI: comparative fit index; RMSEA: root mean square error of approximation; CI: confidence interval; ΔCFI: CFI difference

Regarding measurement invariance of age, the fit indices for the configural, metric and scalar equivalence tests were all considered satisfactory (see Table IV). The chi-square comparison between the configural and metric models, as well as between the metric and scalar models, yielded non-significant differences (*p* > .05) Moreover, the changes in CFI for metric and scalar equivalence were below .010, respectively, which aligns with established criteria [34,35]. These findings indicate that the CERQ-short has measurement invariance across age groups within the study sample.

### Criterion validity

Criterion validity was analysed by testing relationships between CERQ subscales and adolescents’ internalizing problems in terms depression, anxiety, and health-related quality of life. As shown in Table V, partial correlation analyses adjusting for age and gender indicated significant associations between internalizing problems and CERQ-short subscales, except for positive reappraisal. Overall, associations were stronger for subscales indicating self-blame, other-blame, rumination and catastrophizing, compared with acceptance, refocus on planning, positive reappraisal, positive refocusing, and putting into perspective. With regard to quality of life, negative associations were observed for self-blame, acceptance, rumination, and catastrophizing. Positive associations were observed for the use of planning, positive refocusing, reappraisal, and putting into perspective.

**Table V.** Relationships between CERQ-short, HSCL-10 and KIDSCREEN-10.

	1	2	3	4	5	6	7	8	9	10	11
1. KIDSCREEN-10	–										
2. HSCL-10	–.593**	–									
3. Self-blame	–.219**	.431**	–								
4. Other-blame	–.042	.143**	.126**	–							
5. Acceptance	–.098**	.040	.335**	.162**	–						
6. Planning	.138**	.035	.348**	.303**	.426**	–					
7. Positive refocusing	.209**	–.080**	.098**	.246**	.348**	.383**	–				
8. Rumination	–.064**	.257**	.418**	.264**	.428**	.434**	.278**	–			
9. Positive reappraisal	.222**	–.058*	.241**	.249**	.567**	.546**	.467**	.441**	–		
10. Put into perspective	.049*	.140**	.352**	.265**	.438**	.535**	.401**	.315**	.484**	–	
11. Catastrophizing	–.253**	.432**	.468**	.335**	.150**	.240**	.136**	.454**	.154**	.158**	–

* *p* <.05

** *p* < .01.

CERQ-short: Cognitive Emotion Regulation Questionnaire short version; HSCL-10: 10-item Hopkins Symptom Checklist

## Discussion

To the best of our knowledge, this is the first study to document the psychometric properties of CERQ-short for adolescents in Norway. We specifically evaluated the factor structure, internal consistency, age and gender measurement invariance, and criterion-related validity of the Norwegian version of CERQ-short. The findings suggest that the Norwegian adaptation of CERQ-short represents a reliable, quick and effective tool for assessing cognitive emotion regulation strategies among adolescents. All factor loadings were considered excellent, and the data supported the nine-factor model, showing excellent fit. Results from the present study are in line with previous validation studies (e.g. Santos et al. [15], Ireland et al. [37], Betegón et al. [38]), which further solidifies the consistent support for the original nine-factor structure of the 18-item CERQ-short [10]. Other studies, however, have suggested an alternative two-factor structure or two-factor higher-order structure over nine factors [15,30], separating CERQ subscales into adaptive and maladaptive strategies. However, in alignment with another comparable study [38], our findings validated neither a two-factor nor a higher-order two-factor structure. Together with comparable findings from studies with Spanish adolescents [38], this suggest that the nine subscales should not be separated into adaptive versus maladaptive strategies, at least not in the general population. Our findings, however, cannot rule out that emotion regulation strategies may vary between clinical versus non-clinical populations. Indeed, one study did not find support for the nine dimensional structure in a clinical sample of adults with recurrent depression [19], and the two-factor solution has been supported in patients with fibromyalgia syndrome [30]. Whether this is true in clinical populations in Norway needs further investigation.

Consistent with evidence supporting the internal consistency of the nine subscales [10,38], the present data also indicated high reliability for all subscales. Alpha levels were comparable to those of other studies [15,18,37,38], suggesting that the CERQ-short is a reliable instrument for adolescents in Norway. The cross-sectional nature of our data does not warrant estimates of the test–retest reliability; however, other studies measuring the stability of indices over eight weeks indicate adequate values [36].

Results from the series of multi-group CFAs testing configural, metric and scalar invariance showed that the CERQ-short works adequately across gender and different age groups. In other words, results from the nine subscales can be used and compared across boys and girls and between different age groups in early adolescence (i.e. 12 to 16 years of age). These results are consistent with those obtained on other adolescent samples testing the measurement invariance of CERQ-short [15,18]. However, in line with previous studies [18], female adolescents used significantly more self-blame, rumination and catastrophizing compared with male adolescents. Male adolescents, on the other hand, used more positive reappraisal and acceptance. We agree with suggestions from previous studies in that this may imply that female adolescents rely more on emotion-focused regulation [18]. However, in contrast to this interpretation we found female adolescents using putting into perspective to a higher degree than males. In addition, all effects sizes were considered small, reducing the implication of observed differences. Caution should therefore be made when interpreting these findings, and future studies should investigate both within- and between-group effects of cognitive emotion regulation strategies between girls and boys. Our findings underscore the importance of investigating underlying mechanisms of potential gender differences in the use of cognitive emotion regulation strategies.

The criterion-validity of CERQ-short was evaluated by testing relationships with both internalizing problems as indicated by symptoms of anxiety and depression, and with health-related quality of life. The subscales indicating rumination, catastrophizing, self-blame and other-blame correlated moderately to strongly with anxiety and depression as indicated by HSCL-10 scores. This finding is in line with previous research [6,10,36], suggesting that the CERQ-short potentially can identify cognitive emotion regulation strategies associated with increased risk for internalizing problems. However, the positive refocusing subscale was the only one negatively associated with HSCL-10 scores. This is surprising because strategies such as refocus on planning and positive reappraisal have previously been found to be negatively associated with psychopathology [5,6]. These findings should be explored further with other measures of internalizing problems and in clinical adolescent samples with higher levels of anxiety and depressive symptoms. Adolescents’ self-reported use of planning, positive refocusing, positive reappraisal and putting into perspective were all positively associated with quality of life. Thus, using these cognitive emotion regulations strategies appears to improve mental health in terms of higher quality of life in adolescents. This finding is in line with studies on adult samples [22,23]. Our study is among the first to document these associations in an adolescent population. In line with the previous studies investigating relationships to between CERQ and quality of life [22,23], the current results also indicated negative associations between the subscales of self-blame, acceptance, rumination and catastrophizing, and quality of life. Surprisingly, acceptance was also negatively associated with quality of life. The use of these cognitive emotion regulation strategies may be related to a lower quality of life in Norwegian adolescents, and one can speculate whether acceptance to a certain degree overlaps with resignation.

Although the present study has several notable strengths, such as the large and representative population sample of adolescents in Norway, some limitations should be noted. We did not include convergent and divergent validity indices, limiting our analysis of construct validity. Other studies have, however, found support for the convergent and discriminant validity using more suitable indices (e.g. Ireland [37], Araujo [39]). In addition, KIDSCREEN-10 and HSCL-10 were the only scales included to test the criterion validity. Other studies have found that the criterion validity may vary depending on the culture and with the use of different measures of psychopathology [6]. The scales utilized in our study are reliant on self-reporting, which introduces the potential for self-report bias. Subsequent research should incorporate additional objective measures to further assess construct validity. For use of the CERQ-short in clinical contexts, future studies should evaluate the psychometric properties with samples reporting higher levels of psychopathology than in the present sample. Previous research has shown mixed results with regard to the factor structure in clinical adult samples [19], but preliminary evidence supports the original nine-factor model using the full CERQ in adolescent samples with major depressive disorder [2]. Future studies should further explore the factor structure in clinical adolescent samples across psychopathologies. The present study did not test the stability of the subscales over time, and future studies are therefore recommended to document the test–retest reliability in an adolescent sample in addition to internal consistency.

## Conclusion

This study examines the psychometric properties of the CERQ-short for application in population-based research involving Norwegian adolescent samples. The Norwegian adaptation of CERQ-short demonstrated robust psychometric attributes, encompassing a congruent nine-factor model, reliability, measurement invariance, and criterion-related validity. As emotion regulation emerges as a pivotal transdiagnostic feature across various psychopathologies, the imperative to employ thoroughly validated tools becomes even more pronounced. The findings from our investigation endorse the CERQ-short as a promising instrument for such endeavours.
